# Carbuncle

**Published:** 1893-01

**Authors:** J. N. Lowe

**Affiliations:** San Francisco, Cal.


### CARBUNCLE.

J. N. Lowe, Milford, N. J.

In July, 1891, a woman æt. about sixty years, of feeble constitution, came to us, having a very large carbuncle upon the palmar surface of left hand.

The entire palm of her hand was rounded up by it, and it presented in every way, as to pathology and characteristic symptoms, the phenomena of a true carbuncle. Her anxiety and suffering were intense, and it was rapidly making serious inroads upon her vital stamina.

The tumor presented a purplish, livid color, hard and burning. She received Lachesis$^{30}$. This remedy acted with very desirable efficiency, and with the further supplement of Hepar-s-c.$^{6}$ and Silicea$^{30}$, the case progressed to as prompt a cure, and as good a cure, as could possibly be expected.

There was very free suppuration, as the case had been neglected too long before we saw it, and this result was unavoidable. If taken in time, perhaps the morbid process might have been arrested and dissipated by a due attunement of the vital-forces.

Two other cases of carbuncle occurring in men of ordinarily robust constitutions (both in the hands), died of asserted blood poison. These were treated by the old methods of cutting, free incisions, etc., etc., and in one of these cases, at the last, amputation of the hand was resorted to. These three cases of carbuncle all developed within a period of two or three months.

"Boasting is vain," but truth is not; and therefore we feel disposed to challenge any medical or surgical treatment of septicæmia and carbuncle which stands opposed to the genius of corresponding natural laws of cure.

We cannot over-estimate the true value of Lachesis, in the treatment of malignant septic states and conditions, when its pathogenesis presents the characteristics, individual in their totality, of a true similar.

It is truly a most wonderful remedy in its life-conserving power, when the vital constitution of the organic nerves and blood are going to wreck and ruin.

Under given conditions, a true similar will rescue, when all may seem to be lost. All else is vain, useless, and in fact, cruel.

In our case, concisely stated, without Lachesis we might have drifted without a settled purpose, and thus we most surely would have lost our case. Further, I have omitted the fact, that our patient presented symptoms in course, of auto-infection of the blood—septic. At the worst, when she found herself falling to sleep, she felt as if sinking away, and would arouse herself in alarm, fearing she would die if she went to sleep, and worse after sleep.

